# Seed germination demonstrates inter-annual variations in alkaline tolerance: a case study in perennial *Leymus chinensis*

**DOI:** 10.1186/s12870-024-05112-6

**Published:** 2024-05-14

**Authors:** Dandan Zhao, Hongyuan Ma, Shaoyang Li, Wenwen Qi

**Affiliations:** 1 State Key Laboratory of Black Soils Conservation and Utilization, Northeast institute of Geography and Agroecology, Chinese Academy of Sciences, Changchun, Jilin 130102 China; 2 Shandong Key Laboratory of Eco-Environmental Science for Yellow River Delta, Shandong University of Aeronautics, Binzhou, Shandong 256603 China

**Keywords:** Planting year, Seed setting rate, Thousand seed weight, Alkaline stress, Germination

## Abstract

**Background and aims:**

The escalating issue of soil saline-alkalization poses a growing global challenge. *Leymus chinensis* is a perennial grass species commonly used in the establishment and renewal of artificial grasslands that is relatively tolerant of saline, alkaline, and drought conditions. Nonetheless, reduced seed setting rates limit its propagation, especially on alkali-degraded grassland. Inter-annual variations have an important effect on seed yield and germination under abiotic stress, and we therefore examined the effect of planting year on seed yield components of *L. chinensis*.

**Methods:**

We grew transplanted *L. chinensis* seedlings in pots for two (Y_2_), three (Y_3_), or four (Y_4_) years and collected spikes for measurement of seed yield components, including spike length, seed setting rate, grain number per spike, and thousand seed weight. We then collected seeds produced by plants from different planting years and subjected them to alkaline stress (25 mM Na_2_CO_3_) for measurement of germination percentage and seedling growth.

**Results:**

The seed setting rate of *L. chinensis* decreased with an increasing number of years in pot cultivation, but seed weight increased. Y_2_ plants had a higher seed setting rate and more grains per spike, whereas Y_4_ plants had a higher thousand seed weight. The effects of alkaline stress (25 mM Na_2_CO_3_) on seed germination were less pronounced for the heavier seeds produced by Y_4_ plants. Na_2_CO_3_ caused a 9.2% reduction in shoot length for seedlings derived from Y_4_ seeds but a 22.3% increase in shoot length for seedlings derived from Y_3_ seeds.

**Conclusions:**

Our findings demonstrate significant differences in seed yield components among three planting years of *L. chinensis* under pot cultivation in a finite space. Inter-annual variation in seed set may provide advantages to plants. Increased alkalinity tolerance of seed germination was observed for seeds produced in successive planting years.

**Supplementary Information:**

The online version contains supplementary material available at 10.1186/s12870-024-05112-6.

## Introduction

Grasslands, which cover over 40% of the earth’s surface, provide crucial ecosystem services [1, 2]. Approximately 49.3% of the world’s grasslands face degradation problems, which pose significant threats to animal husbandry, ecological security, and sustainable development [3–6]. Saline-alkaline soils are representative of degraded regions throughout the world [7, 8]. Soil salinization and alkalization have been recognized as major environmental threats to agricultural systems, significantly affecting plant growth, physiology, and metabolism [9]. Alkalization reduces community stability in grasslands by reducing species asynchrony and soil nutrient levels, thereby accelerating grassland degradation [10, 11]. To date, research on plant responses to salinity stress induced by NaCl has focused primarily on maintenance of osmotic and ion homeostasis through rapid perception of osmotic and ionic signals and corresponding physiological adjustment [12–14]. However, fewer studies have specially addressed the effects of alkaline stress. Alkaline stress, characterized by high pH and the presence of alkaline salts, has more complex and destructive effects on plants than salinization caused by neutral salts [8, 15–17].

The Songnen grassland, situated in northeast China (121°27′–128°12′ E, 43°36′–49°45′ N), is currently experiencing degradation [18]. Saline-alkalization is leading to significant soil degradation in this region, posing a serious environmental threat with negative effects on sustainable development [19]. *Leymus chinensis* (Trin.) Tzvel, a clonal wild ryegrass with vigorous belowground rhizomes, is the dominant perennial grass species in the Songnen grassland [20]. Because it develops strong rhizomes and adapts well to saline, alkaline, and drought conditions, this species plays an important role in the establishment and renewal of artificial grasslands and in environmental protection [21–25]. *L. chinensis* initiates its inflorescence in the autumn of the year before bloom, this is followed by regrowth in April, heading in May, flowering in June, and seed maturation in July and August. *L. chinensis* experiences the early-vegetative and mid-vegetative stages in August and September, and its growing season ends in late October [22, 26]. *L. chinensis* in the Songnen grassland faces challenges associated with low germination percentage, heading rate and seed setting rate, which hamper its propagation and severely exacerbate the degradation of grassland vegetation. In natural grasslands, *L. chinensis* relies heavily on vegetative propagation for spatial expansion and population renewal [27]. However, rhizomes of *L. chinensis* have a maximum lifespan of four years, and their numbers gradually decrease over successive years. This reduction in rhizome numbers has a significant effect on seed yield [28].

Plants exhibit significant variability in seed production and germination behaviors that can be attributed to various factors, including plant age, growth habitat, and variation among individuals [29, 30]. Thirty-three years of data from *Dacrydium cupressinum* in New Zealand did not support a strong relationship between seed production and variation in environmental factors [31]. Witkowski and Wilson [32] reported that seed production of *Chromolaena odorata* increased over the first 10 years but declined markedly after 15 years. Hampton et al. [28] found that tiller production of perennial ryegrass was optimal at 18–24℃ but that higher temperatures depressed tiller production and seed yield. Pol et al. [33] suggested that perenniality could enable perennial grasses to make large reproductive investments despite harsh environmental conditions. However, little is known about the year-to-year variation in seed yield of *L. chinensis* growing in a finite space within the same habitat [34, 35].

Seed germination and the seedling stage are two critical developmental periods in the plant life cycle during which plants exhibit heightened sensitivity to environmental stresses [36]. Numerous studies have focused on the tolerance of seed germination to saline-alkaline stress [37–39]. Soil salinization delays seed germination process because high soil salt concentrations exceed the critical limit of plant osmotic tolerance, suppressing imbibition and germination [40, 41]. Likewise, alkalinity stress not only triggers the same osmotic stress but also increases the pH, leading to more severe osmotic damage [42]. Giménez-Benavides et al. [43] reported that germination of high-mountain Mediterranean species was highly variable among altitudes, populations, and years, but the results differed among species. However, there is little detailed information on the effects of alkaline stress on germination of *L. chinensis* seeds from different planting years.

In the present study, we measured the seed yield components of *L. chinensis* cultivated in pots for 2–4 years and examined differences in germination, shoot length, and root length of seeds/seedling, with and without alkaline stress. We hypothesized that (1) the seed yield components of the 2nd year plants would be higher than those of the 4th year; and that (2) alkaline tolerance would vary among seeds derived from different planting years. Our results have implications for the effective utilization of *L. chinensis* and the development of appropriate management practices.

## Materials and methods

### Experimental design and sampling

The pot experiment was performed in Changchun, Jilin Province (124°18′-127°02′E, 43°05′-45°15′N) from 2016 to 2019 to study the effect of planting year on seed yield components of *L. chinensis*. This region has a temperate continental climate with a mean annual temperature of 4.9℃ and average annual precipitation of 498 mm.

**Fig. 1:**
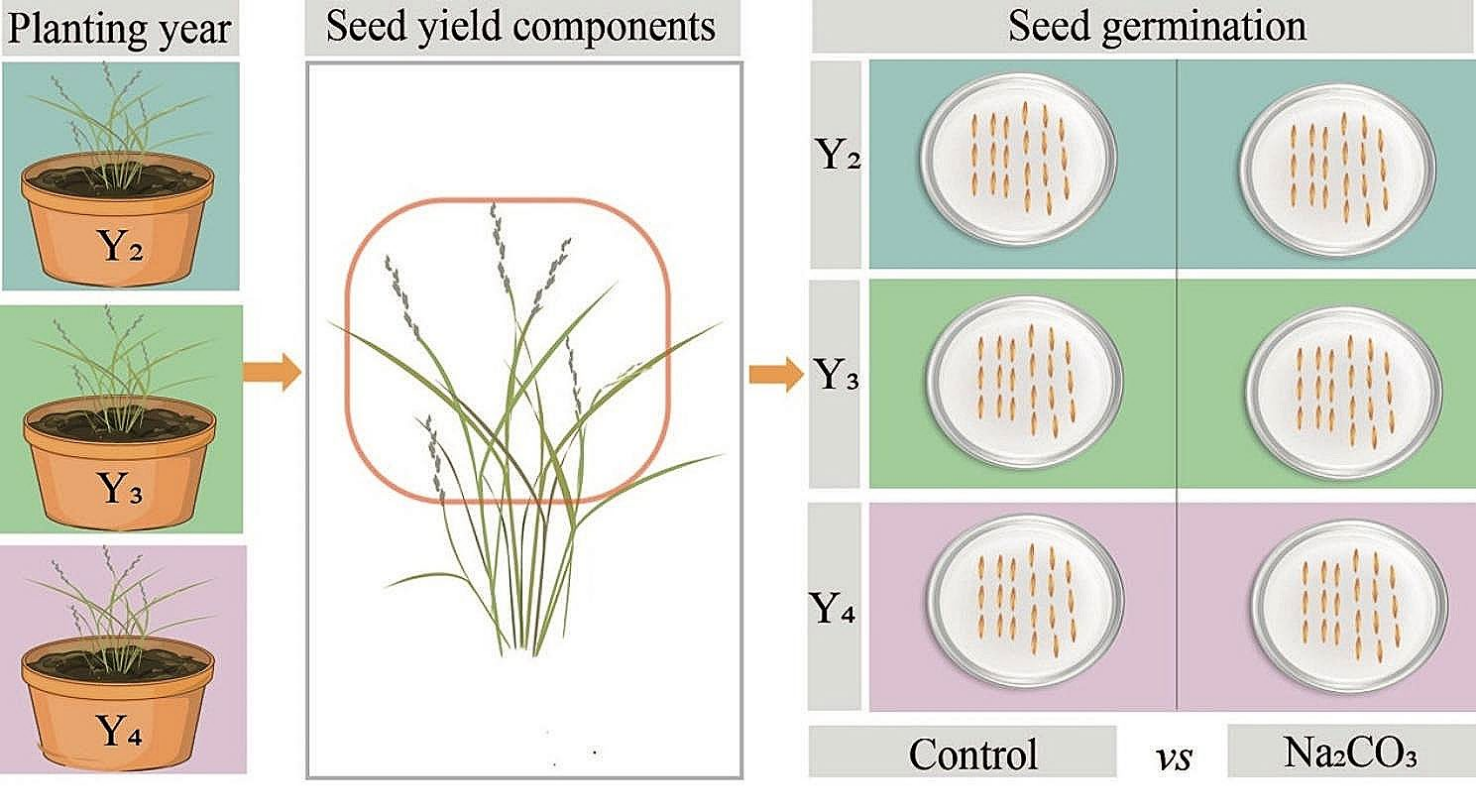
Diagram illustrates the experimental design. Y_2_: transplanted in 2018, 2nd year; Y_3_: transplanted in 2017, 3rd year; Y_4_: transplanted in 2016, 4th year

Plastic pots (diameter and height, 30 cm) were filled with 10 kg of sieved soil that had a pH of 7.12, electrical conductivity of 0.73 dS/m, and concentrations of soil organic carbon, nitrogen, and phosphorus at 2.83%, 1.37 g/kg, and 0.67 g/kg, respectively [44]. *L. chinensis* ‘Dongdi 4’ seedlings of uniform size without offspring ramets were excavated to a depth of 15 cm from the same field site (Figure S1) and transplanted into the plastic pots on 15 May 2016, 15 May 2017, and 15 May 2018 (Fig. 1). Fifteen seedlings were transplanted into each pot, with eight replicates pots per planting year, for a total of 24 pots. Regular watering and weeding were carried out to ensure the normal growth of *L. chinensis*. All plants were grown in the field beneath a transparent polyvinylchloride roof. Belowground irrigation was applied once every two days at 193 ml·pot^− 1^, which was calculated on the basis of 498 mm mean annual precipitation [44]. Concentrations of N, P, and K in the irrigation water were below the limits of detection. Temperature data from Changchun City during the experimental period are provided in Table S2.

Seed yield components were measured for all plants in 2019. At this time, plants transplanted in 2016 were 4 years old (Y_4_), those from 2017 were 3 years old (Y_3_), and those from 2018 were 2 years old (Y_2_) (Table S1). At the stage of seed maturity on 16 August 2019, two spikes were randomly selected from each plot, placed into separate bags, and air-dried in the lab for 3 weeks. Spike length (SL), grain number per spike (GN), and deflated grain number per spike (DN) were measured. Dried plump seeds with glumes from each treatment were divided into 5 groups, each containing 100 grains. Each group was weighed on an electronic, semi-analytical balance (Sartorious AG, Goettingen, Germany). Thousand seed weight (TSW) was calculated as the average weight of the ten groups multiplied by 10. The seed setting rate (SSR) was calculated using the equation [44]:

$${\rm{SSR = }}\frac{{GN}}{{GN + DN}} \times 100\%$$

where *GN* is the grain number per spike and *DN* is the deflated grain number per spike.

### Na_2_CO_3_ treatment and seed germination

In late August 2019, mature seeds of *L. chinensis* were collected from plants of each planting year, air-dried at room temperature, and stored in paper bags at 4℃. The seeds were surface sterilized using 0.1% HgCl_2_ for 10 min and rinses multiple times with distilled water before use. Groups of 20 seeds were sown in 9-cm Petri dishes containing 0.7% (w/v) water agar supplemented with or without (control) 25 mM Na_2_CO_3_; there were three replicate dishes per planting year and treatment. The dishes were incubated in a growth chamber with a 12-h dark, 16℃/12-h light, 28℃ cycle; fluorescent and incandescent white light were provided at 54 µmol⋅m^− 2^s^− 1^. Seed germination was monitored until no new germination occurred over a span of five days. After the germination experiments, five seedlings were randomly selected from each Petri dish for measurements of root and shoot length.

The germination percentage was calculated using the equation:

$${\rm{Germination}}\,{\rm{percentage = }}\frac{n}{N}{\rm{ \times 100\% }}$$

where *n* is the number of germinated seeds at the end of the test and *N* is the total number of seeds tested.

### Statistical analyses

All data were analyzed using R statistical software (R4.2.2). Principal component analysis (PCA) was performed on all traits measured at harvest using the factoextra and factoMinR packages to visualize overall differences in trait variation among the three planting years. From the PCA results, we extracted the explained variance and loadings for each component, which provided insights into the contribution of each variable to its respective component. We repeated the PCA on bootstrapped datasets and then computed the standard deviation from the bootstrapped outputs to obtain the bootstrap standard errors for explained variance, contributions, and loadings. The bootstrapping was performed to estimate the uncertainty of explained variances, loadings, and contributions in the PCA output.

We also used (generalized) linear models to examine the variation in each seed yield component across planting years and to evaluate the effect of planting years on the alkaline tolerance of seed germination. We used (generalized) linear models with a binomial distribution for germination percentage and seed setting rate; a normal distribution for spike length, thousand seed weight, shoot length and root length; and a Poisson distribution for grain number per spike. ANOVA followed by Tukey’s honestly significant difference (HSD) *post hoc test* was used for pairwise multiple comparisons of mean values among planting years and between alkaline stress conditions (R package emmeans).

## Results

### Effect of planting year on seed yield components of *L. chinensis*

**Fig. 2:**
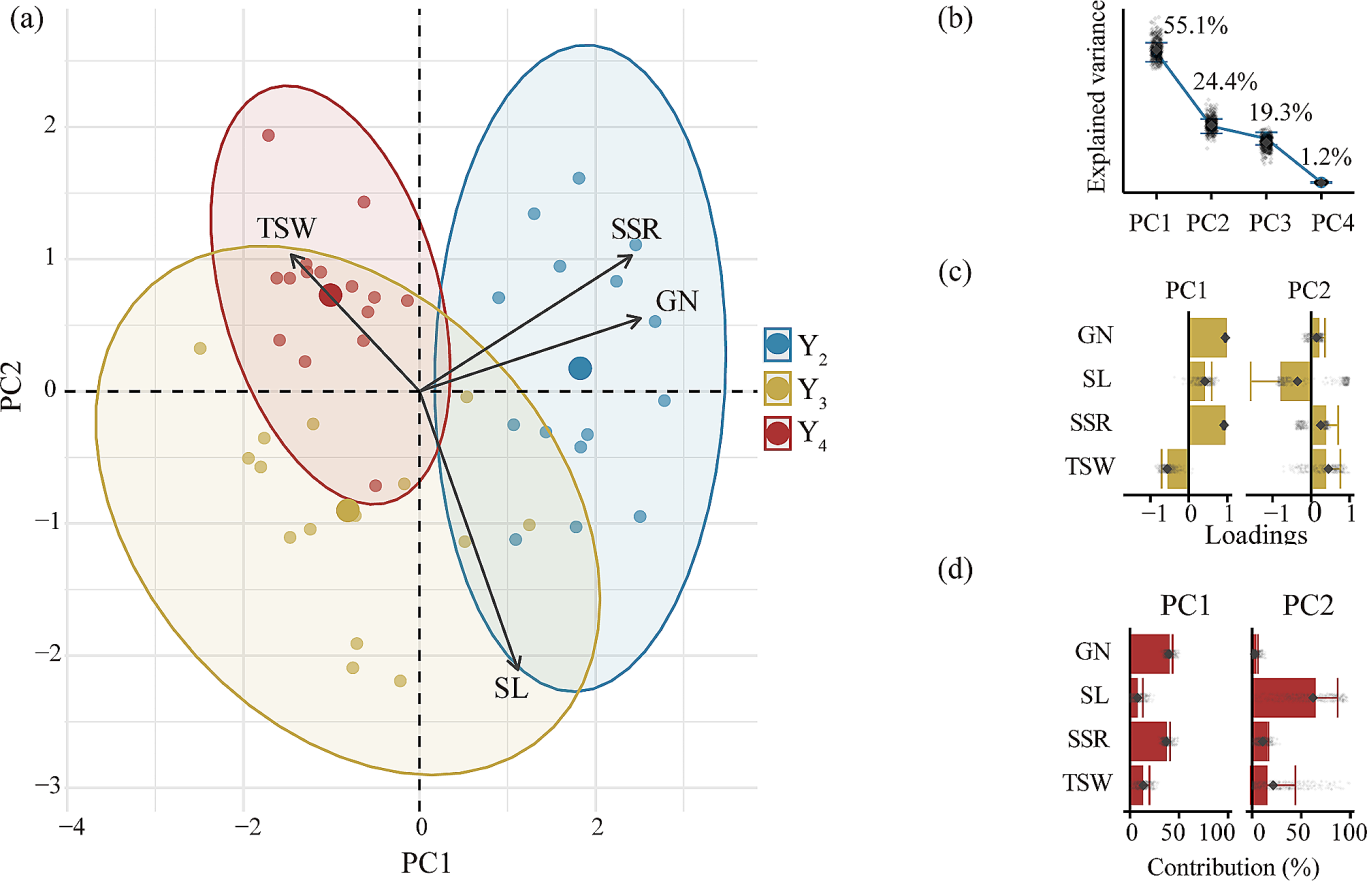
Principal component analysis of seed yield components in different planting years. (**a**) PCA biplot; different colors represent different planting years (Y_2_, transplanted in 2018, 2nd year; Y_3_, transplanted in 2017, 3rd year; Y_4_, transplanted in 2016, 4th year). SL, spike length; GN, grain numbers per spike; TSW, thousand seed weight; SSR, seed setting rate. The three larger points represent the centroids of the distribution for each planting year. (**b**) Percentage of variance explained by the retained principal components (PCs). (**c-d**) Bar plot of the loadings (**c**), and d contributions (**d**) of each variable to PC1 and PC2. The circular symbols in **b** and the bars in (**c**) and (**d**) show the pertinent estimates based on the full dataset. In (**b**-**d**) error bars are centered on the estimates and represent the standard error estimated with the bootstrap procedure (*n* = 500 bootstrap iterations); the small gray diamonds show the estimates of each bootstrap iteration, and the large gray diamonds represent the median of all bootstrap iterations

Together, the first two retained principal components (PCs) explained 79.5% of the variance in the seed yield component dataset (Fig. 2). The first principal component (PC1), explained 55.1% of the variance; it exhibited high positive loadings for GN and SSR and a substantial negative loading for TSW. The second principal component (PC2) explained 24.4% of the variance and had a high negative loading for SL. As shown in Fig. 2a, Y_2_ and Y_4_ plants were clearly separated along the first principal component. The Y_2_ plants had positive values of PC1 associated with higher SSR and GN. By contrast, the Y_4_ plants tended to have negative values of PC1 association with higher TSW.

**Fig. 3:**
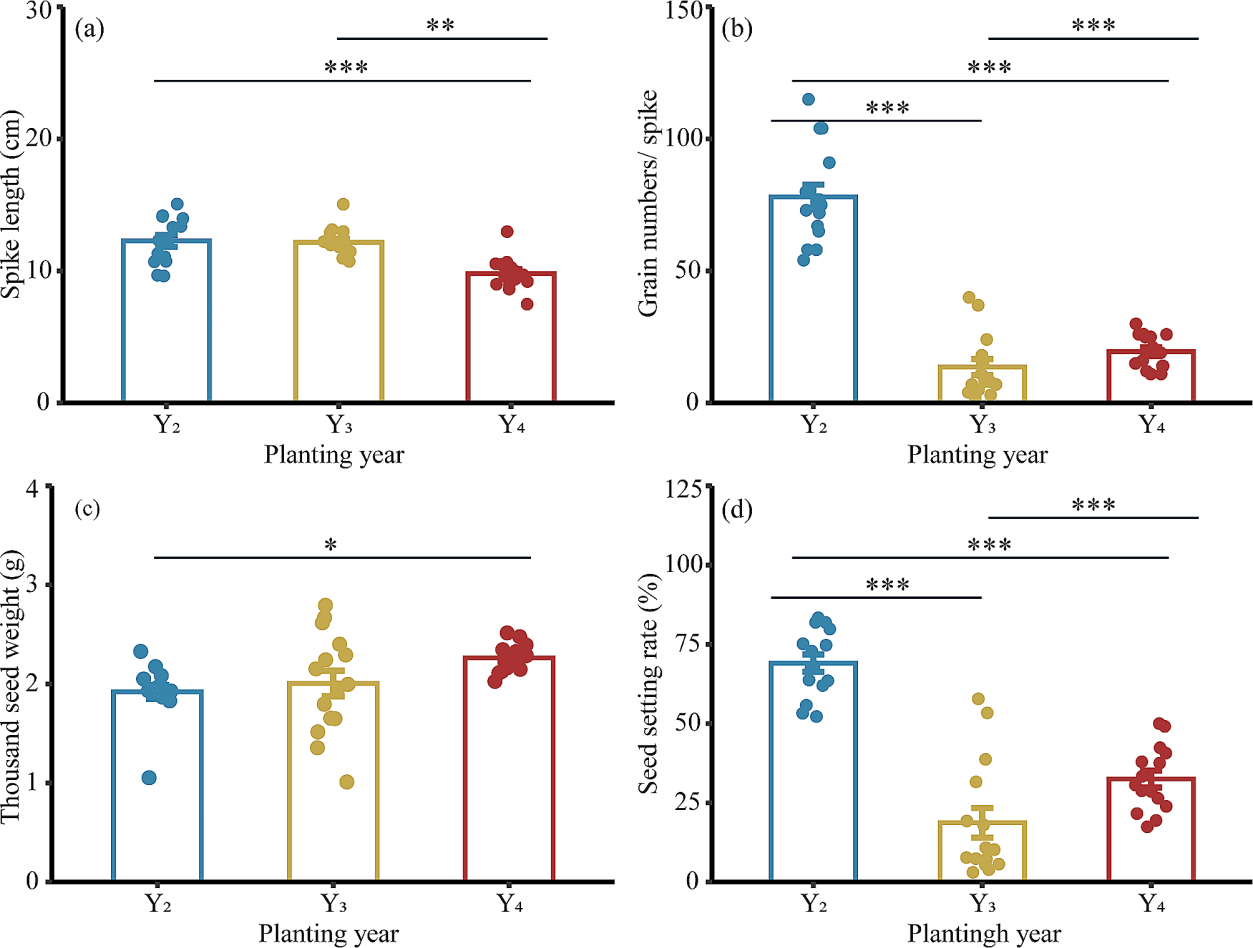
Inter-annual variations in seed yield components of *L. chinensis*. (**a**) Spike length; (**b**) Grain number per spike; (**c**) Thousand seed weight; (**d**) Seed setting rate. Y_2_: transplanted in 2018, 2nd year; Y_3_: transplanted in 2017, 3rd year; Y_4_: transplanted in 2016, 4th year. Asterisks indicate significant differences determined by Tukey’s test (* *P* < 0.05, ** *P* < 0.01, *** *P* < 0.001)

Spike length was significantly shorter in Y_4_ plants than in Y_2_ and Y_3_ plants (Fig. 3a; Table S3). Grain number per spike was significantly lower in Y_3_ and Y_4_ plants than in Y_2_ plants, with reductions of 82.6% and 75.1%, respectively (Fig. 3b). Y_4_ plants had significantly higher thousand seed weight, 17.9% higher than that of Y_2_ plants (Fig. 3c). Seed setting rates were significantly lower in the Y_3_ plants and Y_4_ plants by 72.9% and 52.9%, respectively, compared with the Y_2_ plants (Fig. 3d). Tillers per pot were significantly higher (44.1%) in the Y_2_ plants than in the Y_3_ plants (Figure S2).

### Effects of planting year and alkaline stress on germination and seedling growth of *L. chinensis*

**Fig. 4:**
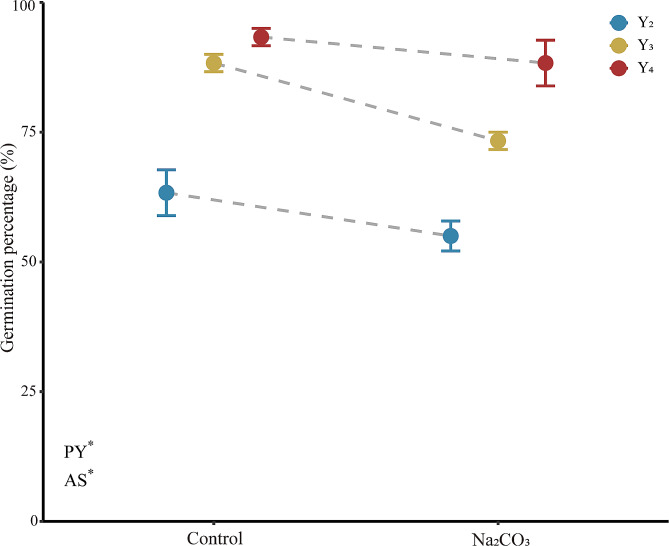
Mean values (± SE) of germination percentages of *L. chinensis* seeds from plants of three planting years (Y_2_, Y_3_, Y_4_) in the presence and absence of 25 mM Na_2_CO_3_. Asterisks indicate that the main effects of both model terms (PY, planting year; AS, alkali stress) were significant at *P* < 0.05. Y_2_: transplanted in 2018, 2nd year; Y_3_: transplanted in 2017, 3rd year; Y_4_: transplanted in 2016, 4th year

Seeds were harvested from Y_2_, Y_3_, and Y_4_ plants for measurement of germination and seedling growth. Both planting year and alkaline stress treatment (25 mM Na_2_CO_3_) had significant effects on the germination percentage of *L. chinensis* seeds (Fig. 4). In the absence of Na_2_CO_3_ stress, the germination percentage was 32.1% lower for seeds from Y_2_ plants than for those from Y_4_ plants. Germination percentage was lower in the presence of Na_2_CO_3_ across all three planting years. Under Na_2_CO_3_ stress, germination percentage was again highest for Y_4_ seeds (88.3%), followed by Y_3_ seeds (73.3%) and Y_2_ seeds (55.0%).

**Fig. 5:**
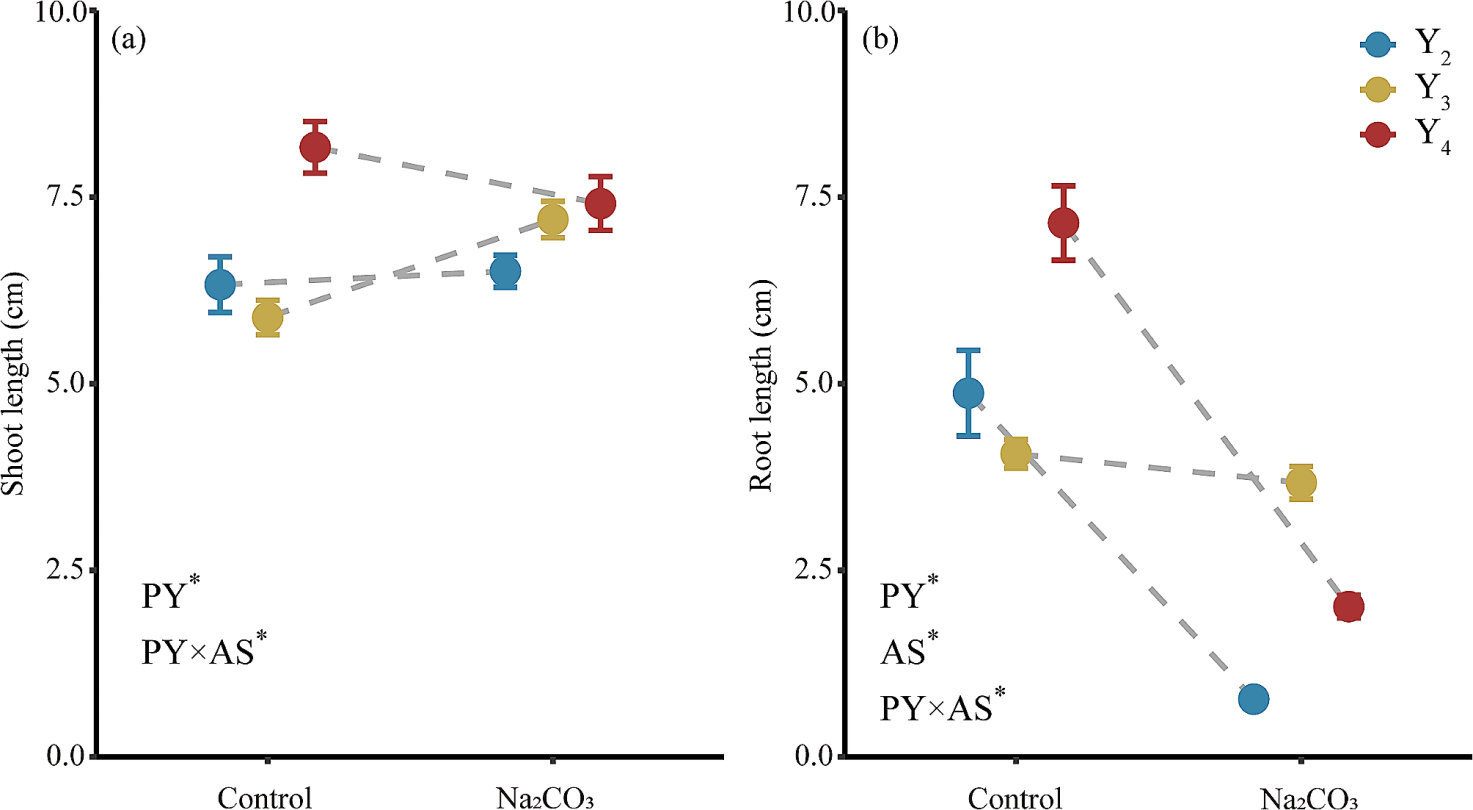
Mean values (± SE) of shoot length (**a**) and root length (**b**) of *L. chinensis* seedlings derived from seeds produced by plants from three planting years (Y_2_, Y_3_, Y_4_), in the presence and absence of 25 mM Na_2_CO_3_. Asterisks indicate main effects and interactions that were significant at *P* < 0.05. Y_2_: transplanted in 2018, 2nd year; Y_3_: transplanted in 2017, 3rd year; Y_4_: transplanted in 2016, 4th year

Shoot lengths of *L. chinensis* seedlings were significantly affected by both planting year and the planting year × alkaline stress interaction (Fig. 5a). Shoots of seedlings derived from Y_4_ seeds were longer than those of seedlings derived from Y_2_ seeds at the same Na_2_CO_3_ concentration. The effects of alkaline stress differed among planting years. Alkaline stress caused a 9.2% reduction in shoot length of the Y_4_-derived seedlings. By contrast, alkaline stress caused a 22.3% increase in the shoot length of Y_3_-derived seedlings but had little effect on that of Y_2_-derived seedlings (6.3 cm vs. 6.5 cm). Planting year, alkali stress, and their interaction all had significant effects on seedling root length (Fig. 5b). Alkaline stress reduced root length of seedlings derived from Y_2_, Y_3_ and Y_4_ seeds by 84.1%, 9.5%, and 71.9%, respectively.

## Discussion

Thousand seed weight of *L. chinensis* increased as the number of years after transplant increased from 2 to 4, although the number of grains per spike decreased. The heavier seeds produced by the Y_4_ plants also showed less pronounced inhibition of germination in the presence of 25 mM Na_2_CO_3_, suggesting that prolonging the duration of pot cultivation had a mitigating effect on alkaline stress in the resulting seeds. These findings align with previous research in which heavier seeds tended to exhibit higher germination percentages than lighter seeds [45]. Our results provide further evidence in support of this notion, as the lighter Y_2_ and Y_3_ seeds tended to have lower germination and reduced shoot and root lengths. However, our findings contrast with those of Fernández-Pascual et al. [46], who found no significant differences in germination between heavy and light seeds of alpine plants. These discrepancies could be attributed to variations in experimental conditions that influence germination behavior. Consistent with previous studies [47, 48], our results demonstrated that seedlings derived from heavier seeds had greater biomass than those derived from lighter seeds, likely due to the presence of greater seed reserves in their cotyledons [49, 50]. Intuitively, heavier seeds would seem to be advantageous because seed mineral reserves should increase as a function of total seed mass [51, 52].

The Y_2_ and Y_4_ plants were clearly separated along the first principal component in Fig. 2. Spike length, grain number per spike, and seed setting rate were all lower in Y_4_ plants than in Y_2_ plants. This result may reflect changes in resource allocation caused by increased plant density and decreased soil nutrient content over multiple years in a limited space [53, 54]. For example, Lou et al. [55] found that seed production gradually decreased in or after the second year of plant growth in the perennial herb *Saussurea nigrescens*. As a plant grows, its demands for nutrients and energy increases, and seed yield declines when supplied nutrients and energy can no longer meet these demands [56, 57]. The planting year of the mother plant has been shown to influence various aspects of seed development and seedling growth in perennial plants species [58]. Here, we observed high inter-annual variation in seed setting rate of *L. chinensis*, consistent with an adaptive seed production strategy. Previous research has suggested that *L. chinensis* exhibits strong plasticity in its reproductive characteristics, particularly in response to inter-annual variations in resource availability [59]. This plasticity enables clonal plants to regulate population stability and promote ecological balance through quantitative adjustments [60, 61].

We observed a decline in the germination percentage of *L. chinensis* under alkaline stress, consistent with previous foundings [62, 63]. The detrimental effects of alkaline stress on germination can be attributed to the effect of osmotic pressure and ion toxicity. The presence of added chlorine ions exacerbates the osmotic stress experienced by the seeds. Moreover, uptake of Na^+^ during seed germination can result in cell Na^+^ toxicity, further inhibiting or delaying the germination process [62–64]. Although alkaline stress inhibited *L. chinensis* seedling growth in the present study, this inhibition was generally less severe for the heavier seeds produced by Y_4_ and Y_3_ plants. The reduced shoot and root lengths under Na_2_CO_3_ stress can be explained by the detrimental effects of high Na^+^ concentrations and high pH stress. Elevated pH levels, particularly in the presence of high sodic salt concentrations, interfere with ion uptake, disrupt intracellular ion balance, damage root cell structure, and ultimately reduce seedling elongation [65, 66]. Variability in germination characteristics has been suggested to be one of the most important survival strategies for species growing under unpredictable environmental conditions [67, 68] and can reduce the risk of seedlings being subjected to poor growing conditions due to the establishment of intense competition hierarchies [69]. This variability helps to reduce the risk of seedlings being subjected to unfavorable growth conditions as a result of intense competition hierarchies.

## Conclusion

Our study revealed clear differences in seed yield components between plants grown for two years after transplant and those grown for four years. The Y_2_ plants had higher seed setting rate and grain number per spike, but the Y_4_ plants had higher thousand seed weight. Seeds obtained from plants grown for four years after transplant showed somewhat less inhibition of seed germination under alkaline stress (25 mM Na_2_CO_3_). A substantial proportion of the inter-annual variation in seed yield components and germination of *L. chinensis* might be due to changes in plant density and/or soil nutrient availability. Further investigations will be required to fully clarify the physiological and molecular mechanisms of the inter-annual variations in germination under alkaline stress.

### Electronic supplementary material

Below is the link to the electronic supplementary material.

Supplementary Material 1

## Data Availability

Data is provided within the supplementary information files.
