# Supplementary material for: Seed germination demonstrates inter-annual variations in alkaline tolerance: a case study in perennial Leymus chinensis
Source: BMC Plant Biol. 2024 May 14;24:397. doi: 10.1186/s12870-024-05112-6 (PMC11092131; doi:10.1186/s12870-024-05112-6)
Supplement: Supplementary file 1 — Supplementary Material 1 [file 12870_2024_5112_MOESM1_ESM.docx]

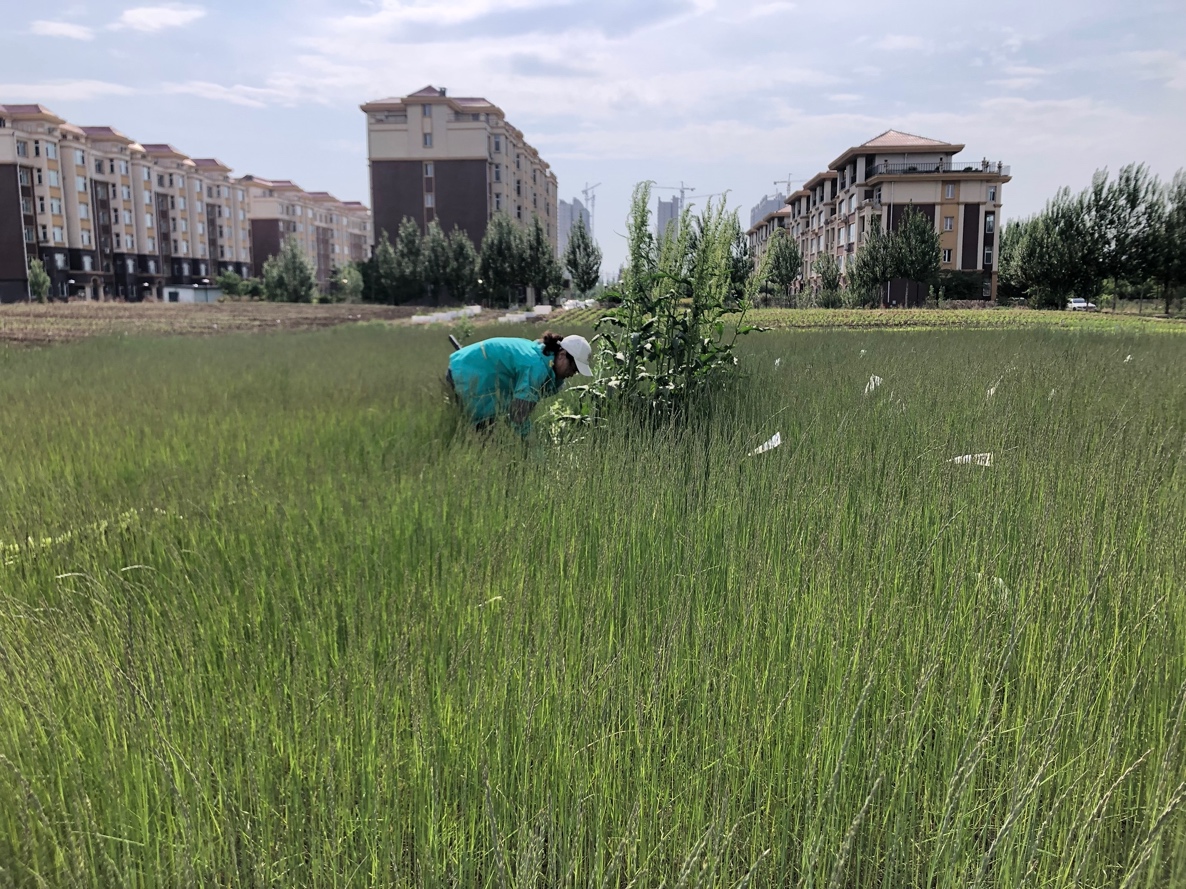


**Figure S1** Field site for transplanting in Changchun.


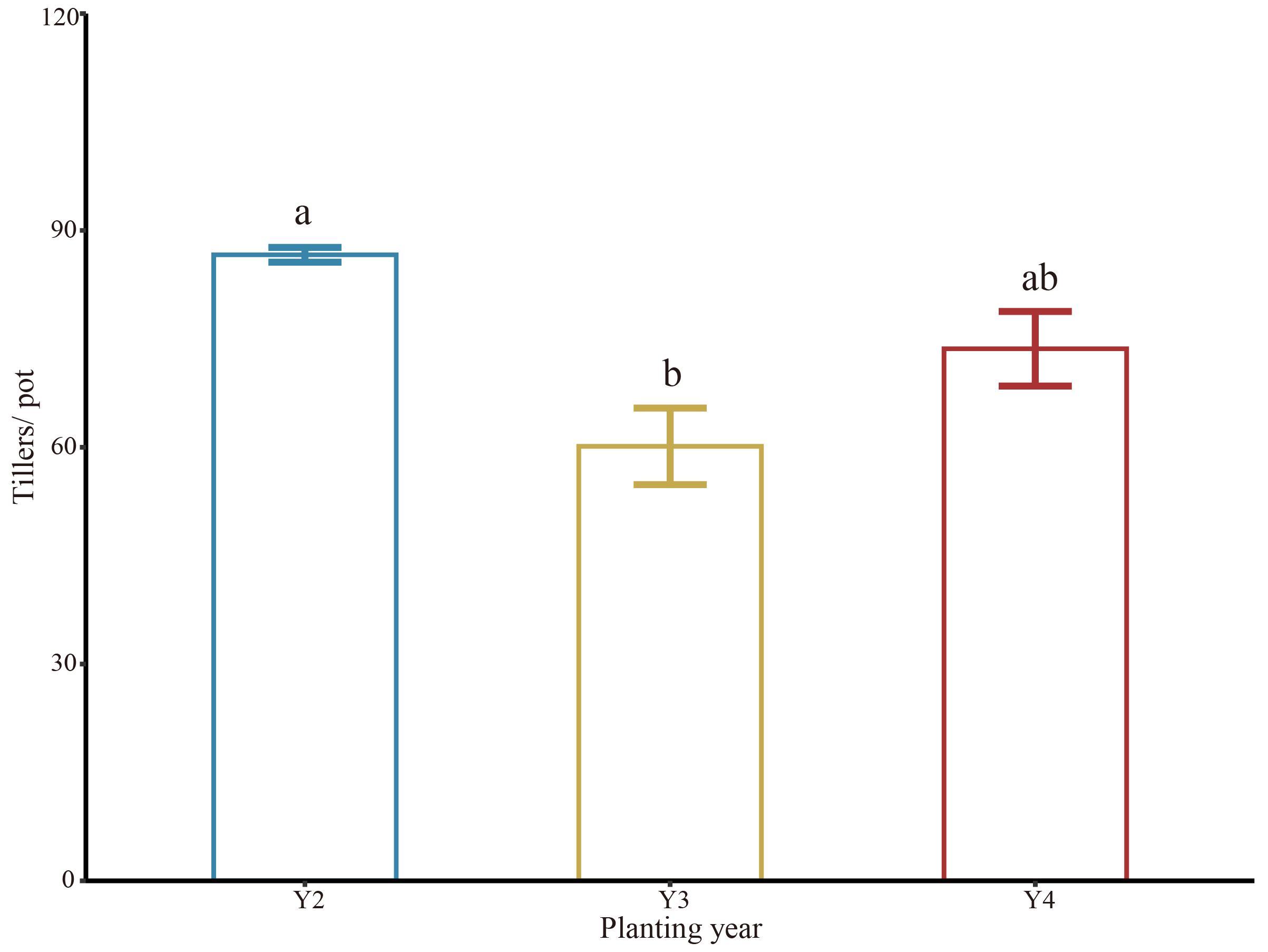


**Figure S2** Effect of planting year on tillers of *L. chinensis* per pot. Y_2_: transplanted in 2018, 2ed year; Y_3_: transplanted in 2017, 3rd year; Y_4_: transplanted in 2016, 4th year. Different lowercase letters indicate significant differences (*p*<0.05) among planting years.

**Table S1** Transplanting time and the corresponding planting year of *L. chinensis*

| Transplanting time | Planting years |
| --- | --- |
| 2016 | Y4 |
| 2017 | Y3 |
| 2018 | Y2 |

**Table S2** Temperature data of Changchun city during the experimental period

| Month | 2016 | | 2017 | | 2018 | | 2019 | |
| --- | --- | --- | --- | --- | --- | --- | --- | --- |
|  | Monthly mean maximum temperature/℃ | Monthly mean minimum temperature/℃ | Monthly mean maximum temperature/℃ | Monthly mean minimum temperature/℃ | Monthly mean maximum temperature/℃ | Monthly mean minimum temperature/℃ | Monthly mean maximum temperature/℃ | Monthly mean minimum temperature/℃ |
| 1 | -12 | -21 | -8 | -18 | -11 | -20 | -5 | -15 |
| 2 | 93 | -13 | -3 | -13 | -7 | -18 | -2 | -12 |
| 3 | 7 | -3 | 6 | -5 | 5 | -7 | 8 | -4 |
| 4 | 14 | 3 | 17 | 4 | 16 | 5 | 16 | 3 |
| 5 | 22 | 11 | 23 | 11 | 23 | 11 | 24 | 12 |
| 6 | 26 | 16 | 26 | 15 | 27 | 17 | 26 | 15 |
| 7 | 29 | 20 | 29 | 20 | 30 | 22 | 29 | 21 |
| 8 | 28 | 18 | 26 | 18 | 26 | 18 | 25 | 18 |
| 9 | 22 | 13 | 22 | 11 | 21 | 11 | 24 | 12 |
| 10 | 11 | 1 | 13 | 2 | 14 | 3 | 15 | 3 |
| 11 | -1 | -10 | 1 | -7 | 4 | -6 | 2 | -7 |
| 12 | -5 | -13 | -8 | -17 | -6 | -16 | -7 | -16 |

**Table S3** Inter-annual variations in seed yield components of *Leymus chinensis*

| Years | SL | GN | TSW | SSR |
| --- | --- | --- | --- | --- |
| Y_2_ | 12.3±0.4a | 78.0±4.7a | 1.9±0.1b | 69.1±2.7a |
| Y_3_ | 12.2±0.3a | 13.6±3.0b | 2.0±0.1b | 18.7±4.7c |
| Y_4_ | 9.8±0.3b | 19.4±1.7b | 2.3±0.04a | 32.5±2.6b |

Y_2_: transplanted in 2018, 2nd year; Y_3_: transplanted in 2017, 3rd year; Y_4_: transplanted in 2016, 4th year. SL: spike length; GN: grain numbers per spike; TSW: thousand seed weight; SSR: seed setting rate. Different lowercase letters indicate significant difference (*p* < 0.05) among different planting years.
